# Effect of Deep Brain Stimulation on Swallowing Function: A Systematic Review

**DOI:** 10.3389/fneur.2020.00547

**Published:** 2020-07-17

**Authors:** Huiyan Yu, Kazutaka Takahashi, Lisa Bloom, Samuel D. Quaynor, Tao Xie

**Affiliations:** ^1^Department of Neurology, Beijing Hospital, National Center of Gerontology, Beijing, China; ^2^Department of Neurology, The University of Chicago Medicine, Chicago, IL, United States; ^3^Department of Organismal Biology and Anatomy, The University of Chicago, Chicago, IL, United States; ^4^Speech and Swallowing Service, The University of Chicago Medicine, Chicago, IL, United States

**Keywords:** deep brain stimulation (DBS), dysphagia, swallowing function, subthalamic nucleus (STN), globus pallidus interna (GPi), post-subthalamic area (PSA), Parkinson's disease, movement disorders

## Abstract

The effect of deep brain stimulation (DBS) on swallowing function in movement disorders is unclear. Here, we systematically reviewed this topic by searching keywords following PICOS strategy of problem (swallowing or swallow or dysphagia or aspiration) and intervention (deep brain stimulation, or DBS) in the PubMed and Web of Science in English in April 2020, with comparators [subthalamic nucleus (STN), globus pallidus interna (GPi), ventralis intermedius, (ViM), post-subthalamic area, or caudal zona incerta (PSA/cZi); ON/OFF DBS state/settings, ON/OFF medication state, Parkinson's disease (PD), dystonia, tremor], outcomes (swallowing function measures, subjective/objective) and study types (good quality original studies) in mind. We found that STN DBS at usual high-frequency stimulation could have beneficial effect (more so on subjective measures and/or OFF medication), no effect, or detrimental effect (more so on objective measures and/or ON medication) on swallowing function in patients with PD, while low-frequency stimulation (LFS) could have beneficial effect on swallowing function in patients with freezing of gait. GPi DBS could have a beneficial effect (regardless of medication state and outcome measures) or no effect, but no detrimental effect, on swallowing function in PD. GPi DBS also has beneficial effects on swallowing function in majority of the studies on Meige syndrome but not in other diseases with dystonia. PSA/cZi DBS rarely has detrimental effect on swallowing functions in patients with PD or tremor. There is limited information on ViM to assess. Information on swallowing function by DBS remains limited. Well-designed studies and direct comparison of targets are further needed.

## Introduction

Dysphagia, or impaired swallow function, is one of the two major causes of mortalities in Parkinson's disease (PD) (along with falls related to the loss of balance). Dysphagia usually does not respond well to dopaminergic medication treatment (1, 2). Although deep brain stimulation (DBS) has significant beneficial effects in PD patients with motor fluctuation, dyskinesia, or medication refractory tremor (3–7), it has less benefits in axial symptoms of balance, speech, and swallowing function. Some studies even raise concerns about worsening of the axial symptom after DBS, particularly with long-term DBS at the usual high-frequency stimulation (HFS) (8–13), while axial symptoms have been found to predict the mortality of PD patients with STN DBS (14). Low-frequency stimulation (LFS) has been reported to have beneficial effect on axial symptoms in patients with freezing of gait (FOG) at usual HFS (15–18). Most common DBS targets to treat PD are STN (subthalamic nucleus) or GPi (globus pallidus interna) (3–7). They both have a similar effect on motor function of PD, but different effects in non-motor symptoms, such as cognitive function and depression, with different extents in medication reduction after the surgery as well (5, 19). GPi also seems to have a better outcome on axial symptoms, particularly after more than 2-year stimulation compared to STN (12).

The effect of DBS on swallowing function has not been well-studied across various movement disorders and targets. There was a retrospective study on the effect of unilateral STN vs. unilateral GPi on swallowing function in PD patients, which demonstrated a better swallowing function in penetration–aspiration (PA) scores on the videofluoroscopic swallow study (VFSS) in GPi compared to STN at medication OFF status, although there was a difference in baseline swallowing function between these two groups (20). LFS of STN was found to have beneficial effect on dysphagia compared to HFS in patients with FOG refractory to usual HFS of STN (16, 17). DBS targeting the post-subthalamic area and caudal zona incerta (PSA/cZi) was thought to be associated with fewer side effects compared to ventralis intermedius (ViM) or STN (21), including the swallowing function (22–24). GPi DBS has also been used to treat various dystonia (25–28), including Meige syndrome (29–32), and its effect on the swallowing function is also of interest to review compared to that in PD.

Besides diseases and targets, ON/OFF DBS state and stimulation frequencies, ON/OFF medication state, outcome measures for swallowing function (subjective questionnaires or scales vs. objective assessments, such as VFSS), and study designs (randomized double blind vs. open label retrospective or prospective) could also affect the swallowing function.

There was only one review article specifically focusing on the effect of DBS on swallowing function comparing different targets in the literature, mainly on unilateral GPi to STN DBS in patients with PD (33), which was published about 7 years ago. Therefore, it is necessary to have a comprehensive review with updated information on the effect of DBS on swallowing function covering various targets and movement disorders to reflect recent advances in the field, which will help guide our clinical practice in applying DBS for movement disorders.

## Methods

**Figure 1:**
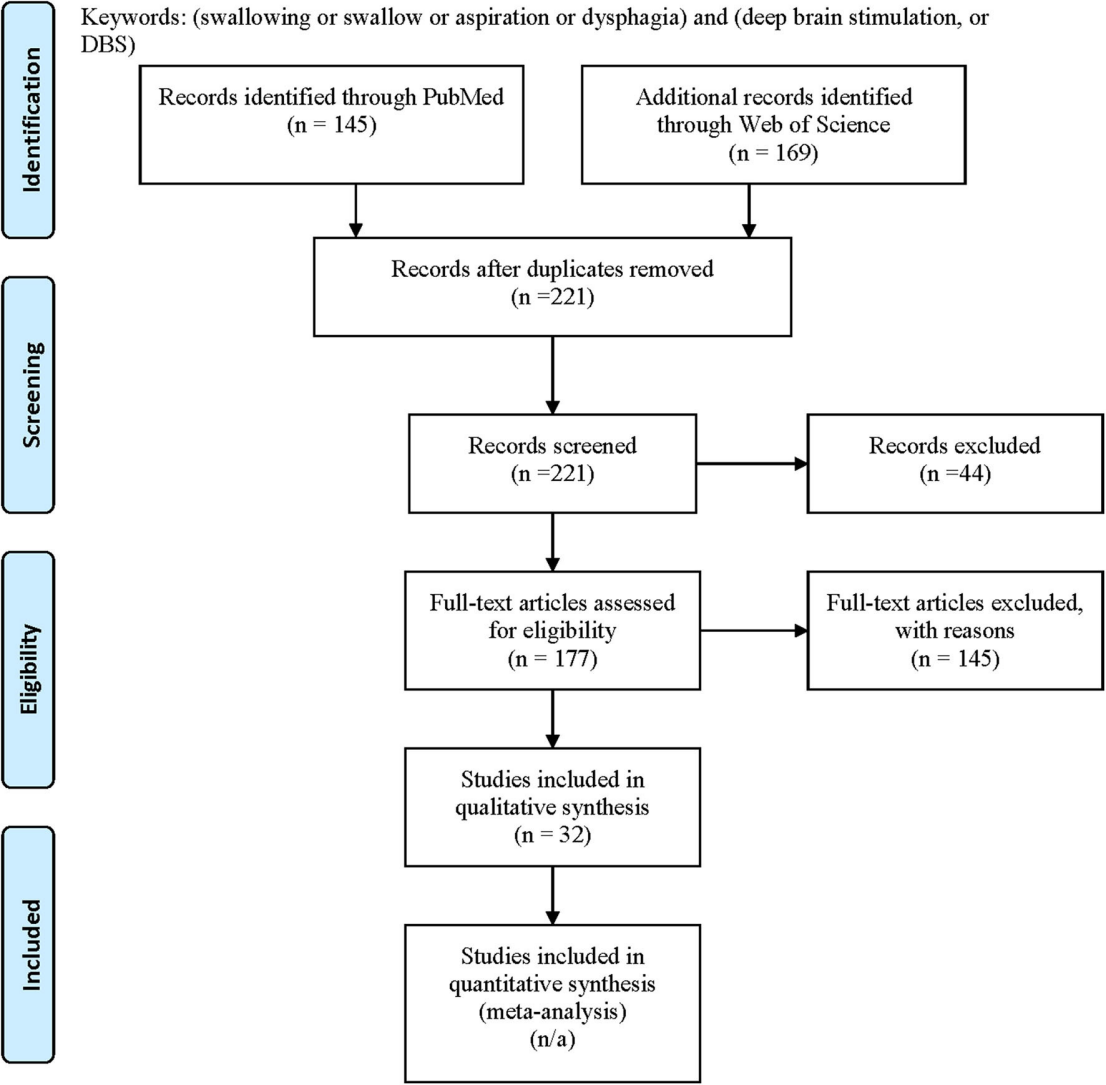
PRISMA flow diagram: literature search and selection with numbers of articles at each stage.

We systematically searched the PubMed and the Web of Science in April 2020 for all available publications in English by keywords following PICOS concepts: problem = (dysphagia or swallowing or swallow or aspiration) and intervention = (DBS or deep brain stimulation) to include all pertinent articles, with comparators [subthalamic nucleus (STN), globus pallidus interna (GPi), ventralis intermedius (ViM), post-subthalamic area or caudal zona incerta (PSA/cZi), ON/OFF DBS state/settings (ON/OFF) medication state; Parkinson's disease (PD), dystonia, tremor], outcomes (swallowing function measures, subjective/objective) and study types (good quality original studies) in mind during the search. We followed PRISMA guideline for systematic review, and the flow chart of the literature search and selection process of the review is depicted in Figure 1 (34, 35). A total of 145 publications were found from PubMed and 169 from Web of Science. After removing the duplicate entries, screening was performed to narrow down to 177 articles by excluding reviews, comments, viewpoints, author responses, letters, book chapters, single case reports with insufficient information, and meeting abstracts. Then the full texts were assessed, and we removed studies without clear outcome measures on swallowing function by DBS. We finally identified 32 unique articles. We included DBS studies targeting STN, GPi, ViM, or PSA/cZi on patients with PD, various dystonia (including Meige syndrome), and essential tremor (ET), and compared swallowing function measures (subjective vs. objective) at ON/OFF DBS state under different settings (including stimulation frequencies), or post-operative to pre-operative baseline, at ON/OFF medication state. Basic demographics and types of study designs (retrospective vs. prospective, open vs. blind) were also taken into consideration in assessments.

## Results

Each pertinent publication is listed in detail in Table 1, with information on references, diseases, DBS targets, basic demographics, study designs (randomized double blind vs. open label retrospective or prospective), outcome measures (subjective vs. objective measures) on swallowing functions at ON/OFF DBS or post-operational vs. pre-operational state under different DBS settings (if available) and ON/OFF medication state, and major conclusions. Among the 32 articles identified, 22 articles were on PD patients, with 19 targeting STN, 3 targeting GPi, and 3 targeting PSA/cZi, as some studies were targeting more than one target. There were six articles on Meige syndrome and five on non-Meige dystonia or dyskinesia (including primary generalized dystonia, segmental dystonia, and cerebral palsy), all targeting GPi. There was only one article on ET targeting PSA and none on ET targeting ViM on swallowing function. The majority of the studies used HFS of 125–210 Hz, but two studies used LFS of 60 Hz (16, 17). The assessments included subjective measures, such as swallowing questionnaires or scales, and objective measures, such as VFSS and fiberoptic endoscopic evaluation of swallowing (FEES).

**Table 1:** The effect of DBS on swallowing functions.

**References**	**Diseases, targets (STN vs. GPi vs. PSA/cZi) and side (Unil vs. Bil)**	**Age at study, and/or disease duration (Mean ± Std, unless noted) (Years)**	**Design and assessment**	**DBS settings**	**Outcome (ON/OFF medication, ON/OFF DBS)**
(8)	PD, Bil STN	Age of 57.7 ± 8.4 yo, PD duration of 14.4 ± 5.8 years	Retrospective chart review, in 27 PD, unknown M/F ratio, assessed dysphagia after DBS, as adverse effect, in a mean of 30 months after DBS, unclear ON or OFF medication status when and how the dysphagia was assessed. Medication dose was also reduced by 39% at 12 months and 30% at 30 months.	Unknown	At least three patients developed worsening or new dysphagia after the DBS, in a mean of 30 months post-operation (post-op).
(16)	PD, Bil STN	Age of 64.0 ± 8.0 yo and PD duration of 12.9 ± 4.9 years, s/p Bil STN-DBS for 4.4 ± 4.9 years.	Prospective, sequence randomized, crossover, double-blind study in seven PD patients with refractory FOG at HFS of 130 Hz and ON medication, each received VFSS under DBS of 130, 60 Hz, or OFF DBS, all ON medication. The laryngeal PA events and a swallowing questionnaire were assessed. UPDRS-III motor score, axial subscore, tremor subscore, and FOG by a questionnaire and stand-walk-sit test were also assessed. DBS condition with the least FOG (60 Hz) was maintained for 6 weeks on average, and patients were assessed again then (at 60 Hz). Changes in measurements between the 60 Hz and 130 Hz at initial assessment, and between 60 Hz of 6 weeks apart were analyzed, with swallowing function as primary and the remainder as secondary outcomes. Changes between other DBS conditions were also explored.	Amplitudes: Rt 3.1 ± 0.4 V; Lt 3.2 ± 0.4 V. Pulse widths: Rt 81.4 ± 14.6 μs; Lt 90.0 ± 24.5 μs. Frequencies: 130, 60 Hz, OFF Configurations: 13 active contacts on monopolar and one active contact on bipolar configurations.	Compared with the routine 130, 60 Hz significantly reduced aspiration frequency by 57% on VFSS and reduced the perceived swallowing difficulty by 80% on questionnaire. It also significantly reduced FOG, overall axial symptoms and parkinsonism. The benefits at 60 Hz stimulation persisted over the 6-week assessed.
(17)	PD, Bil STN	68.5 ± 5.9 yo, PD duration of 14.2± 5.7 years and DBS duration of 3.5 ± 4.0 years	A prospective, sequence randomized, crossover, double-blind study, PD patients with DBS refractory FOG at 130 Hz and ON medication were randomized to sequences of 130, 60 Hz, or OFF DBS to assess swallowing function by VFSS, FOG severity (stand–walk–sit test and FOG questionnaire) and motor function (UPDRS-III) at initial visit (V1) and follow-up visit (V2, after being on 60 Hz stimulation for an average of 14.5 months), in usual ON medication state. The frequency of aspiration events, perceived swallowing difficulty and FOG severity at 60 Hz compared with 130 Hz at V2, and their corresponding changes at V2 compared with V1 at 60 Hz were set as primary outcomes, with similar comparisons in UPDRS-III and its subscores as secondary outcomes.	Amplitudes: L: 3.0 ± 0.4 V, R: 2.9 ± 0.3 V Pulse widths: L: 76.0 ± 24 μs, R: 68.0 ± 14 μsFrequencies: 130, 60 Hz, OFFConfigulrations: 20 leads on monopolar, 2 leads on bipolar; 16 on dorsal and 6 on ventral active contacts.	All 11 participants completed V1 and 10 completed V2. They found benefits of 60 Hz compared to 130 Hz in reducing aspiration frequency, perceived swallowing difficulty, FOG severity, bradykinesia and overall axial and motor symptoms at V1, with persistent benefits on all of them except dysphagia at V2, with overall decreasing efficacy when comparing V2 to V1.
(20)	PD, Unil STN or GPi	PD duration 11.21 ± 5.21 years for STN, 12.11 ± 4.15 years for GPi	Retrospective chart review, 33 PD, M/F 28/5, 14 on Unil STN, 19 on Unil GPi, before and 6 months after DBS on PA score of VFSS and SWAL-QOL scores. The assignment on the target was not randomized.	Unknown	PA scores significantly worsened in STN but not in GPi at ON DBS state and ON medications state. No change in SWAL-QOL scores before and after the DBS for either group. However, the GPi group had worse swallowing function at the baseline than the STN before the DBS.
(22)	PD, Bil cZi	Age 49–71 yo, median 62 yo; disease duration 6.1 ± 2.8 years.	Open label, prospective longitudinal study, 8 PD patients, M/F 6/2, Bil cZi, swallowing function before and 6 and 12 months after DBS on any of the swallowing parameters, assessed by FEES and self-assessment questionnaire. Pre-op patients were examined ON (1.5 times of the ordinary levodopa equivalent) and OFF meds. Post-op ON medication, with ON/OFF DBS.	Unknown	No clear-cut effect of DBS at 6 and 12 months on any of the swallowing parameters except the pre-swallow spillage that was slightly worsened ON DBS at 12 months post-op. Overall no negative effect on swallowing function.
(23)	PD, Bil cZi	Median 53 yo for PD and 54 yo for controls.	Open label, prospective, longitudinal study, 9 PD, M/F 7/2, compared to 9 controls in SWAL-QOL scale and VA scale before (ON and OFF meds) and 12 months after Bil cZi DBS (ON medications, ON/OFF DBS)	Unknown, except 125–160 Hz	No significant differences between the pre- or post-op scores. No difference between PD and controls. cZi not negatively affecting the swallowing QOL.
(24)	PD, Bil cZi	Median 57 yo, with median disease duration of 6 years	Open label, prospective longitudinal study on 14 PD patients with Bil cZi, M/F 12/2, extending their previous report on swallowing function using FEES, before (ON medications, 1.5 × of the original dose) and 12 months after DBS at ON medications (original dose) and ON DBS On vs. OFF DBS state, on PA scale, secretion severity scale, premature spillage and pharyngeal residual.	Unknown, except 125–160 Hz	cZi DBS was found not to have a negative impact on swallowing safety, with no changes on PA, pharyngeal residual or premature spillage. Speech function noted to be worse.
(25)	DYT6, Bil GPi	Age at DBS 8–57 yo. Disease duration before DBS 2–19 years. Length follow up after the DBS: 1–16 years 4 months.	Retrospective multiple centers case serials of medical records in 14 DYT6 patients, 9F, 5M, with BFMDS and the sub-scores as the outcome measures at a median of 4 year 10 months post-surgery compared to that before the surgery	Stimulationfrequency 90–180 Hz at their last follow up visit. Details unknown.	No improvement in swallowing and speech function in 10/14, and some improvement in 4/14.
(26)	Primary general or segmental dystonia, Bil GPi	20 neurostim (13 M) and 20 sham stim (14 M), age 40.5 ± 13.5 and 38.4 ± 13.8 yo, respectively; disease duration 21.8± 8.1 and 17.2 ±7.5 years, respectively	A randomized, controlled trial, with 40 patients randomly assigned either to neurostim or sham stim for 3 months. Primary end point was the change form baseline to 3 months on BFMDRS. Subsequently all patients received open label neurostim; blinded assessment was repeated after 6 months of active treatment.	Neurostim: 3 months 3.2/122.2/139.5 (V/μs/Hz) 6 months: 3.2/123.7/135.7 Sham stim: 3 months: N/A 6 months: 3.2/131.3/132.8 unknown contacts, xyz (mm): 20 to 21/2/−2 to −6	Significantly benefit in dystonia on neurostim than sham stim at 3 months. No improvement in swallow and speech after 6 months neurostim.
(27)	Dystonia, Bil GPi	19 patients (12 M); Age at surgery 47.3 ± 12 yo; mean disease duration 13.7 ± 10.9 years, with isolated generalized (*n* = 10), segmental (*n* = 4) or cervical dystonia (*n* = 5) and chronic GPi DBS for up to 16 years (11 ± 2.6 years) for follow up.	Retrospective analysis in 19 patients, analyzing BFMDRS at baseline, short-term (range 3–36 months) and long-term follow-up (range 93–197 months). Quality of life and mood were evaluated using the SF-36 and Beck Depression Index questionnaires.	Unknown	GPi DBS is a safe and efficacious long-term treatment for dystonia with sustained effects on motor impairment and disability, accompanied by a robust improvement in mood and quality of life. The most common stim-related side effects were dysarthria (*n* = 4), swallowing difficulties (*n* = 1) and bradykinesia (*n* = 2), which were all partially reversible with adjustment of stimulation settings.
(28)	Dyskinesic CP, Bil GPi	Age 30 ± 6.8 yo at study, about 4 years after the surgery	Eight patients with dyskinesic CP, s/p Bil GPi were openly assessed by BFMDR Scale. Subjective impression of the extent of postoperative change as well as gait, speech and swallowing performances (by fiberoptic laryngoscopy) also assessed during ON/OFF DBS.	1.2–3.8 V/90–210 μs /all 120–180 Hz, except one 5 Hz. Active contacts: 0, 8, 0, 8, 0, 8, 3, 7, 2, 5, 0, 4, 0, 4, 1, 5.	No change in objective assessment of speech and swallowing function after DBS compared to baseline, but patients reported subjective improvement.
(29)	Meige Syndrome, Bil GPi	Age at surgery 64.5 ± 4.4 yo, mean PD duration 8.3 ± 4.4 years	Retrospective study, in 12 patients with Meige syndrome, M/F 6/6, followed up to 78 mon after Bil GPi. BFMDR speech and swallowing subscore in short –term (4.4 ± 1.5 months) and long-term (38.8 ± 21.7 months) follow-up.	Rt 2.4–5.0 V/60–210 μs/130–210 Hz, Lt 2.2–4.9 V/ 90–210 μs/130–210 Hz. Most of them on bipolar or monopolar	BFMDR speech and swallowing subscore improved by 44 and 64% respective in short—term and long-term assessment.
(30)	Meige syndrome, Bil GPi	Age 58.0 ± 7.8 yo, duration: 8.7 ± 7.6 years.	Retrospective study in 11 cases, unknown M/F, Meige syndrome, Bil GPi DBS, on BFMDRS, f/u for more than 12 months (mean 23.1 ± 6.4 months).	3.4 ± 0.6 V/ 133.6 ± 576.4 μs/ 143.1 ± 38.1 Hz (last follow up), xyz 21.6/2.8/– 4, unknown contacts	Improved by 68.4% for speech and swallowing subscore at 12 months after DBS. No difference between 12 and 24 months
(31)	Meige syndrome, Bil GPi	Mean age 58.5 yo, disease duration 12.5 years	Open label, prospective follow up study, in 6 cases of Meige syndrome, M/F 2/4 Bil GPi, BFMDRS assessed before and after DBS compared to the baseline scores in short-term (3 months) and long-term (6–60 months) post-op follow up.	3.4–4.1 V or 2.5–3.2 mA, 117–120 μs, 130–160 Hz; 9 on double monopolar.	Speech/swallowing subscore improved by 49% in short-term and 39% in long-term assessment.
(32)	Meige syndrome,	Age: 41.5 yo, Bil GPi	Retrospective study, 40 patients (M/F 16/24), Bil GPi with Meige syndrome. Motor functions were assessed using the BFMDRS and subscores. The severity of patients' dystonia was evaluated before surgery and at follow-up DBS.	All 40 patients received monopolar stimulation with the average voltage of 2.6 ± 0.8 V, pulse width of 90.0 ± 21.1 μs, and frequency of 88.0 ± 21.3 Hz.	At 6, 12, and 24 months after surgery, the BFMDRS subscores of eyes, mouth, speech, and swallowing and mouth movement were significantly better. The overall improvement rate was 83%
(36)	PD, Unil and Bil STN	Median 61 yo (41–72), disease course unknown	Open label, prospective study, 11 patients (5 Unil and 6 Bil STN) evaluated before and 6 and 12 months after DBS, using self-estimation on a VA scale (11 patient) and FEES (8 patient) including PA scale, secretion severity scale, pre-swallow spillage, pharyngeal residue and clearance, ON/OFF DBS, at ON medication	Unknown	Subjectively improved with DBS on self– assessments, but no improvement on objective FEES.
(37)	PD, Bil STN	Age of 66.6 ± 6.2 yo, with 11.6 ± 5.7 years of PD	Open label study, 18 patients, M/F 8/10, Bil STN, with clinical swallowing impairments, evaluated at pre- and 6 month post-DBS, using VFSS comparing ON DBS ON medication to pre-op ON medication (though with more LED than post-op) on oropharyngeal transit times, speed of tongue movement and laryngeal elevation delay time and dysphagia scale score, and comparing ON DBS to OFF DBS at ON medication post-op as well.	Unknown	STN-DBS may not significantly improve overall swallowing function, but may improve tongue movement and laryngeal elevation
(38)	PD, Bil STN	Age of 57.3 ± 4.7 yo; disease duration 13.0 ± 2.4 years	Longitudinal prospective descriptive study, 10 PD, M/F 10/0, Bil STN DBS, clinical assessment of anamnesis, Functional Oral Intake Scale, and clinical swallowing function before and 6 months after the DBS	Unknown DBS configurations and parameters, or medication status (but no changes in levodopa equivalent dose pre and post-DBS).	No change in swallowing function 6 months after DBS compared that before DBS.
(39)	PD, Bil STN and Bil GPi	27 PD, 14 with 16.8 ± 6.2 years of PD for STN, 13 with 15.1 ± 10.2 years of PD for GPi. 27 age and gender matched healthy control subjects.	Randomized, double-blind, longitudinal study, with matched healthy controls, in 14 PD with Bil STN and 13 PD with Bil GPi, M/F 25/2, assessed before (OFF/ON medication) and 6 months after DBS (OFF/ON medication and OFF/ON DBS) on self-scaled and externally-scaled jaw peak velocity.	Mean amplitude 3.28 V, with 70% of the patients on 90 μs of pulse width (60, 120, and 150 in two subjects each), and 71% on 185 Hz (the rest was between 130 and 150 Hz). xyz for STN: 12/−4/−4 mm; xyz for GPi: 20–21/2/−4 mm. No specific contact settings available.	OFF medications: DBS in STN worsened while GPi improved jaw velocities by self-scale 6 months after DBS compared to baseline. ON medications: velocities in STN still worse than the baseline, but no difference in GPi. Similar results also revealed by external scale. No benefit of STN or GPi on jaw velocity in PD compared to the best medication therapy. STN could even be harmful
(40)	PD, Bil or Unil STN	Age of 57.9 ± 9.6 yo, disease duration 8.3 ± 3.7 years	Retrospective study, in 85 PD, M/F 52/33, Bil (51) or Unil (34) STN DBS, assessed before (ON/OFF meds) and 4.9 years after DBS (ON/OFF medication and ON/OFF DBS) on UPDRS-II (swallowing) and UPDRS-III (speech)	2.9–3.1 V/86–88 μs/163–174 Hz	Long-term STN DBS failed to improve swallowing and speech (swallowing and speech parameters even worsened with DBS).
(41)	PD, Bil STN,	Age of 63.4± 6.7 yo (10 M), disease duration unknown. DBS duration at least 6 months post-op. Healthy control (HC) age of 68.1± 10.7 yo (16 M)	Controlled, randomized, double blind, crossover trial, 15 PD patients were assessed with DBS Stim OFF, STN-DBS, STN + SNr- DBS. Patients and 32 age-matched HC were examined clinically and by FEES to evaluate the swallowing function. The primary end point was the assessment of residues, secondary endpoints were penetration/aspiration, leakage, retained pharyngeal secretions, drooling, and assessments of the patient's self-perception of swallowing on a VA scale.	The tip of the electrodes >4.5 mm below to AC-PC line. Various DBS parameters. All at HFS 125–130 Hz.	Eleven completed the study. Four dropped out from STN/SNr Stim due to side effects. Compared with HC, PD patients showed significantly more pharyngeal residues in Stim OFF and both DBS modes. Residues or aspiration events were found in 80% of the patients under STN-Stim. STN + SNr-Stim had no additional positive effect on swallowing function compared to STN-DBS.
(42)	PD, Bil STN	74 yo, male, PD of 14 years	Case report. The patient experienced stridor and dysphagia with pulmonary restriction and aspiration, which started 4 months after Bil STN DBS, and significantly improved when the DBS was OFF.	Initial left: monopolar(unknown exact contact) 1.6 V/90 μs/130 Hz; right: monopolar, 1.6 V/60 μs/130 Hz. Final left: bipolar, 1.5 V/60 μs/160 Hz; right: bipolar 2.1 V/60 μs/160 Hz	All his symptoms improved after DBS turned off or adjusted to bipolar settings, suggesting that the initial dysphagia was related to suboptimal placement or programming.
(43)	PD, Unil STN (R), followed by Unil GPi (L) DBS	PD since 29 yo, age of 51 yo had Rt STN DBS sub- optimally placed.	Case report. A 62 yo male PD, VFSS after Bil DBS (Rt STN first, followed by Lt GPi), with dysphagia confirmed by VFSS, which improved when the suboptimal Rt STN was OFF. Reassessed with improvement after DBS parameters optimized.	Rt STN, 3.8 V/90 μs/135 Hz, Lt GPi, 2.9 V/120 μs/135 Hz	Marked, immediate improvement with optimizing DBS settings compared to previous DBS settings.
(45)	PD, Bil STN	2 women (62 and 76 yo) and 12 men (mean 59 yo; range 41–75 yo)	Open label, prospective study, 14 patients, M/F 12/2, Bil STN, VFSS pre- and 3- and 12-mon post-DBS, ON/OFF DBS and ON/OFF medication, with DHI being assessed as well at each time.	Unknown	Subjective but no objective improvement in swallowing function. Specifically, there was a trend toward improved swallowing response for solid intake and oral preparation of thin liquid in OFF meds with ON/OFF 12 mon later. The remaining swallowing parameters showed no change regardless of the DBS or medications states. DHI revealed improved self- perception of swallowing 3 and 12 months post-op compared with the baseline.
(46)	PD, Bil STN,	Age of 61.2 ± 6.2 yo at surgery, the duration of PD 16.7 ± 4.4 years	Open label, prospective study, in 36 PD, M/F 22/12, pre-op (ON and OFF meds) and 12 and 24 months post-op. Post-op ON medications (but with reduced dosage) and ON DBS, comparing with pre-op OFF/ON medications baseline, on salivation, swallowing and sensory complaints in UPDRS-II corresponding items.	3.2 ± 0.4 V; 63.3 ± 9.5 μs; 136 ± 14.8 Hz at 12 mon; 3.3 ± 0.3 V; 65.0 ± 11.3 μs; 136.1 ± 12.5 Hz at 24 mon. Most of them on mono polar setting	Salivation, swallowing and sensory complaints ameliorated by ON DBS with reduced meds compared to pre-op OFF medication, but no changes compared to pre- op ON medication status. (Levodopa equivalent dosage 60 and 59% reduction at 12 and 24 months, respectively)
(47)	PD, Bil STN	PD onset age of 49.3 ± 10.2 yo. PD duration at time of surgery 135.3 ± 68.7 months	Retrospectively collected data for a prospective study in 18 PD, M/F 11/7, ON medication, before and 20 months after DBS (medication reduced by 50%), comparing swallowing before vs. after DBS and ON vs. OFF DBS using VFSS and “New Zealand Index for Multidisciplinary Evaluation of Swallowing Subscale One” for qualitative and “Logemann-MBS-Parameters” for quantitative evaluation.	0.5–6.0 V/60–120 μs/65–180 Hz. Configurations: 26 of the leads were monopolar; the rest were bipolar, double bipolar and double monopolar.	Postoperatively, medications reduced by 50%. No clinically relevant effect of DBS on swallowing was observed using qualitative parameters. However, quantitative parameters found significant changes of pharyngeal parameters with ON DBS as compared to pre-op and OFF DBS mostly with fluid consistency. They concluded that DBS modulates the pharyngeal phase but has no clinically relevant influence on overall deglutition.
(48)	PD, Bil STN	Age of 58.0 ± 6.5 yo, disease duration 10.9 ± 4.7 years	Open label study in 20 PD, M/F 15/5, Bil STN DBS, The frequency and severity of gastrointestinal symptoms (including dysphagia) based on a structured gastrointestinal dysfunction questionnaire also assessed, at OFF medication state.	Unknown configuration but 1–2 V/60 μs/130 Hz	DBS improves gastric motility and symptoms. Gastrointestinal dysfunction questionnaire improved by > 50% with dysphagia 3 months post-op, at OFF medication but ON DBS
(49)	PD, Bil STN	Age of 67.5 ± 6.5 yo, disease duration 15.2± 4.8 years. DBS median duration of 13 months	Open label study in 34 PD (M/F 23/11), OFF medication state, ON DBS compared to OFF, in subjective VAS for non-motor symptoms, including dysphagia in the study	Unknown	DBS improved the dysphagia.
(50)	Meige syndrome, Bil and Unil GPi	Median ages 61 (41–72) yo, and median duration 6.5 (1–13) years.	Retrospective review of videos and charts in 6 cases, M/F 4/2, 1 Unil and 5 Bil GPi, evaluated 6 months and 12 months for UDRS and BFMDR including speech and swallowing function.	1.5–3.5 V/60–450 μs/10–185 Hz. Configurations: 9 monopolar, 1 bipolar, and 1 double monopolar	Swallowing and speech did not improve in this cohort.
(51)	Dystonia (Meige syndrome and crural dystonia), Bil GPi	Mean age 42.8 (30–67) yo, mean disease duration 18.5 (12–25) years	Retrospective analysis, 11 segmental dystonia (9 Meige syndrome, 2 crural type dystonia), M/F 3/8, Bil GPi, assessed pre-op and post-op 6–12–24–36 months, by BFMDRS	3.2 ± 0.5 V/150 ± 60 μs/130 Hz. Monopolar configuration in all patients, with ventral contacts in all except two patients.	Speech and swallowing function improved significantly at 6 months and 36 months post-op.
(52)	ET, PSA, 19 patients with ET had Unil and 2 had Bil PSA DBS.	Age of 63.6 ± 14.8 yo, ET duration of 20.3± 13.7 years.	A prospective study in 21 patients (M/F 14/7) with ET were included in this study for the efficacy and safety of PSA DBS. Eight patients presented a postoperative mild dysphasia that regressed within days to weeks.	The mean stim parameters: 2.5 ±0.8 V, 61.4 ±6.0 μs, 165± 21 Hz, and monopolar stim in 78% leads.	Effective and safe in tremor control, with transiently mild dysphagia regressed within days to week

*STN, subthalamic nucleus; GPi, globus pallidus interna; ViM, ventralis intermedius; PSA, post-subthalamic area; cZi, caudal zona incerta; M, male; F, female; yo, year-old; FOG, freezing of gait; s/p, status post; Bil, Bilateral; Unil, unilateral; HFS, high frequency of stimulation; LFS, low frequency of stimulation; VFSS, videofluoroscopic swallow study; FEES, fiberoptic endoscopic evaluation of swallowing; PA, penetration-aspiration; UPDRS, Unified Parkinson's Disease Rating Scale; V, voltage; L, left; R, right; SWAL-QOL, swallowing related quality of life; VA, visual analogue; DYT6, dystonia by the THAP1 mutation; BFMDS, Burke-Fahn-Masden Dystonia Rating Scale; neurostim, neuronal stimulation; stim, stimulation; SNr, substantia nigra reticular; DHI, Dysphagia Handicap Index; UDRS, Unified Dystonia Rating Scale; ET, essential tremor*.

We summarized the result as below, based on the diseases and targets.

### PD With STN DBS

STN DBS in patients with PD can have no effects (36–38). Kitashima et al. reported no improvement in swallowing function in 18 PD patients assessed by VFSS at ON medication state 6 months after the bilateral STN DBS (37). Olchik et al. found no change in swallowing function 6 months after bilateral STN DBS in 10 PD patients assessed by anamnesis, functional oral intake scale, and clinical swallowing function (38).

STN DBS in patients with PD can also have detrimental effects on the swallowing function. STN DBS impaired the jaw opening and closing velocities by scales 6 months after DBS compared to baseline regardless of ON/OFF medication state in a randomized double blind study in 14 patients with bilateral STN DBS (39). Xu et al. did not find any improvement on swallowing function based on the item on Unified Parkinson's Disease Rating Scale (UPDRS) Part II in 85 PD patients assessed on an average of 4.9 years after STN DBS (mixed unilateral and bilateral STN DBS) at ON/OFF medication state (and the swallowing function was even worse ON DBS) (40). Troche et al. reported significantly worse in the PA score of VFSS in 14 PD 6 months after unilateral STN DBS at ON medication state (20). Kraus reported that at least three patients developed worsening dysphagia or new dysphagia after bilateral STN DBS in a group of 27 PD patients during a mean of 30 months follow-up, based on the assessment for adverse effect, with unclear medication state though (8). Add-on stimulation of substantia nigra reticular (SNr) to STN did not have beneficial effect (41). Worsening of the dysphagia could be related to the suboptimal placement of the DBS electrodes or suboptimal programming in some cases, as turning off or reprogramming of the DBS made the swallowing symptoms better or go away in these cases (42, 43).

Some studies even reported beneficial effects on the swallowing function but mostly at OFF medication status, on subjective measures, or at LFS. Ciucci et al. reported significantly improved pharyngeal composite score and transit time by VFSS in ON DBS compared to OFF DBS at OFF medication status in 14 PD patients assessed at least 3 months after STN DBS (44). Kulnef et al. reported a subjective improvement in a self-assessment of swallowing function, but not on objective FEES, at ON DBS compared to OFF DBS at ON medication state in 11 PD patients 6 and 12 months after STB DBS (a mixed bilateral and unilateral DBS) (36). A similar result was also reported by Silbergleit et al. in 14 PD patients 3 and 12 months after bilateral STN DBS assessed by VFSS who found subjective but not objective improvement in swallowing function at ON/OFF medication state (45). Zibetti et al. found improved salivation and swallowing function in 36 patients with PD and bilateral STN DBS at 12 and 24 months after DBS at OFF medication state but no difference at ON medication compared to the pre-operational state (although the levodopa dosage was also reduced then) (46). Lengerer et al. reported no clinically relevant influence of DBS on swallowing function using qualitative parameters in 18 PD patients with bilateral STN DBS, but quantitative parameters found improved pharyngeal parameters with ON DBS compared to preoperative condition or OFF DBS, mostly with fluid consistency (47). Krygowska-Wajs et al. reported a 50% improvement on dysphagia on the gastrointestinal dysfunction questionnaire in 20 PD patients, assessed 3 months after bilateral STN DBS at ON DBS but OFF medication state (48). Wolz et al. studied 34 PD patients at a median of 13 months after the bilateral STN DBS and found improved dysphagia in subjective visual analog (VA) scale at ON DBS compared to OFF DBS and OFF medication state (49). Xie et al. reported acute and short-term improvement of objective and subjective swallowing function on PD patients with bilateral STN DBS in randomized double blind crossover studies under LFS (60 Hz) compared to those under HFS (130 Hz) in patients with HFS and medication refractory FOG at ON medication state (16, 17). However, the long-term (more than a year) benefit of LFS on the swallowing function was not demonstrated (17).

### PD With GPi DBS, and Compared to STN as Well

Troche et al. performed a retrospective chart review in 33 PD patients, with unilateral GPi DBS in 19 and unilateral STN DBS in 14 patients, looking at PA score of VFSS and patient-reported swallowing-related quality of life (SWAL-QOL) before and 6 months after DBS (20). PA scores significantly worsened in STN but not in GPi DBS assessed at ON medication state. No change in SWAL-QOL score was found before and after the DBS in either group of patients. The GPi group patients had worse swallowing function than the STN group at baseline. Robertson et al. randomized the PD patients to STN or GPi in double-blind study in 14 PD with bilateral STN and 13 PD with bilateral GPi, assessed before (OFF medication vs. ON) and 6 months after DBS (OFF medication vs. ON medication and OFF DBS vs. ON DBS) on self-scaled and externally scaled jaw peak velocity (39). At OFF medication state, DBS in STN worsened, while GPi improved the jaw velocities after DBS compared to baseline. At ON medication state, the velocities in STN were worse than the baseline, but no difference in GPi. The authors concluded that there was no benefit of STN or GPi on jaw velocity in PD compared to the best medication therapy, and that STN could even be harmful.

### PD With PSA/cZi DBS

The swallowing function of eight PD patients with bilateral cZi DBS was assessed before and after DBS by FEES and questionnaire (22). There was no clear-cut effect of DBS at 6 and 12 months on any of the swallowing parameters except for the pre-swallow spillage, which was slightly worse in the ON stimulation state 12 months after DBS, although the medication was cut down by one-third post-operatively. Sundstedt et al. found no significant difference in SWAL-QOL score and VA scale score 12 months after the DBS at ON medication state in nine PD patients with bilateral cZi (23). Sundstedt et al. also did a prospective longitudinal study on 14 PD patients with bilateral cZi, extending their previous report on swallowing function, before and after DBS at ON medications and ON DBS vs. OFF DBS state by FEES (24). They found that cZi DBS did not have a negative impact on swallowing function, with no changes on PA scores, pharyngeal residual or premature spillage, although the medication was cut down by one-third post-operatively.

### Dystonia and Meige Syndrome With GPi DBS

Bilateral GPi DBS has been shown to improve the swallowing function in majority of the studies in patients with Meige syndrome, as demonstrated by improved Burke–Fahn–Masden Dystonia Rating Scale (BFMDRS) speech and swallowing scores in 12 patients who followed up to 38 months on average (29), in 11 patients who followed up for 23 months on average (30), in 6 patients who followed up to 60 months (31), and in 40 patients who followed up at 6, 12, and 24 months after surgery (32). There was one study by Limotai et al. in six patients with Meige syndrome, with one unilateral and five bilateral GPi, evaluated 6 and 12 months after DSB for Unified Dystonia Rating Scale (UDRS) and BFMDR speech and swallowing function, but they did not find improvement in speech and swallowing function in this cohort (50). Bilateral GPi also has been used in patients with 11 non-Meige dystonia patients and 9 Meige syndrome patients (51), with significantly improved swallowing and speech scores in BFMDR up to 36 months after the DBS. Bilateral GPi also has been used in primary generalized dystonia and segmental dystonia patients (25–27), and dyskinetic cerebral palsy patients (28), but no changes or just slightly worsening in speech and swallowing function after DBS compared to baseline were reported.

### ET With ViM or PSA/cZi DBS

There is no specifically designed study on the evaluation of dysphagia in ET by ViM or PSA/cZi DBS, although transient mild dysphagia after the DBS implantation surgery was reported, which usually resolved within several weeks (21, 52).

## Discussions

The majority of the studies were open label, retrospective or prospective, small-size studies, with subjective and/or objective assessments of swallowing function, at ON DBS compared to OFF DBS and ON/OFF dopaminergic medication state. There were only a few prospective randomized double blind studies (16, 17, 39), a few on comparing different targets (20, 39), and a few on comparing different frequency stimulations (16, 17). Most studies used bilateral targets although some were unilateral or mixed targets, as bilateral DBS is more likely to affect the axial symptoms, including dysphagia. Some of them were not fairly compared, as there were reduced dopaminergic medications post-operatively. Although the medications probably would not have a major impact on the objective swallowing functions (1, 2), beneficial effect of dopaminergic medication was also reported in a small proportion of patients (53). Taking dopaminergic medications could also affect the subjective measure with overall improvement of the parkinsonism. Therefore, it probably could explain why some studies showed improved swallowing function at subjective measures but not objective measures at ON medication state, and why the beneficial effect of DBS is more appreciated at OFF medication state or less appreciated at ON medication state.

We found that STN DBS at usual HFS could have beneficial effect (more so on subjective measures of scales, questionnaires, or swallowing item in UPDRS-II, and/or OFF medication state), no effect, or detrimental effect (more so on objective measures of VFSS or FEES, and/or ON medication state) on swallowing function in patients with PD. The effect of LFS stimulation on FOG has been consistently reported positively by many studies, as summarized in a review article (18). However, there have been only a few studies addressing its effect on dysphagia. Two studies of randomized double blinded crossover prospective studies in the short- and long- term effects did find significant benefit of LFS on acute and short-term studies (16, 17), but not the long-term benefits (17), although the long-term effect remains unclear given the small sample size and sub-clinical dysphagia in participants, which could limit the power to detect the potential difference. These studies were conducted at ON medication state in bilateral STN DBS patients with refractory FOG to HFS; hence, the beneficial effect should not necessarily be generalized to the whole PD population.

GPi DBS seems more likely to improve the swallowing function or process compared to the STN DBS, more so at OFF medication state (20, 39). In contrast to STN DBS, GPi DBS does not have detrimental effect on swallowing function or process at ON medication state (20, 39). Even though the non-matched baseline swallowing function in the two groups, and the retrospective and non-randomized design in assigning the targets could all affect the interpretation of the favorable PA scores in unilateral GPi compared to STN DBS (20), similar results were also obtained in a randomized, double-blind study comparing the effect of bilateral GPi to bilateral STN DBS on jaw velocity (39), suggesting that GPi DBS is probably more favorable than STN DBS in overall swallowing function for PD patients, particularly at OFF medication state. Although there is no benefit of STN or GPi DBS on swallowing function in PD compared to the best medication therapy (at ON medication state), STN DBS could even be harmful at ON medication state, based on limited studies available so far.

Targeting GPi seemed to have positive results on Meige syndrome in the majority of the studies (29–32). One of the possibilities behind the benefit is the direct effect on the pharyngeal and laryngeal dystonia by GPi, which could help to improve dysphagia symptoms. There was no study on using STN in Meige syndrome and other dystonia on dysphagia. Hence, it is not certain if targeting STN would have similar benefit, as STN has also been found to be beneficial to dystonia in PD (54). There is no beneficial effect of GPi DBS on dysphagia in patients with primary generalized dystonia, segmental dystonia, and dyskinesic cerebral palsy patients, and there rarely is worsening effect either (25–28).

The PSA and cZi are relatively new targets. They have the potential to provide more efficient stimulations but fewer side effects due to their anatomic characteristics, with the fibers from both the basal ganglia and cerebellar merging together at the PSA/cZi area, and studies so far found that PSA/cZi DBS rarely has a detrimental effect on swallowing functions in patients with PD or tremor (21, 55). There has been limited information on the effect of ViM DBS on swallowing function to assess so far.

In summary, we found that STN DBS at usual HFS could have beneficial effect (more so on subjective measures and/or OFF medication state), no effect, or detrimental effect (more so on objective measures and/or ON medication state) on swallowing function in patients with PD, while LFS of STN could have beneficial effect on swallowing functions in PD patients with FOG refractory to HFS. GPi DBS could have a beneficial effect (regardless of medication state, and subjective or objective measures), or no effect (more so at ON medication state), but no detrimental effect (in contrast to STN DBS, even at ON medication state) on swallowing function in PD, suggesting that GPi DBS could be probably more favorable than STN DBS in overall swallowing function for PD patients, particularly at OFF medication state. GPi DBS also has beneficial effects on swallowing function in the majority of the studies on Meige syndrome but no beneficial effect on swallowing function in other dystonia. Stimulation of PSA/cZi rarely has detrimental effect on swallowing functions. The effect of ViM on swallowing function in ET patients is too limited to assess. Overall, most of them are retrospective, open label, small-size studies, with medication reduction post-operatively. There are only a few randomized, double blind studies, a few on direct comparisons among targets or between stimulation frequencies. The overall evidence levels of these studies are low, ranging from IV to III. Information on swallowing function by DBS remains limited. Well-designed studies and direct comparison of targets and stimulating parameters are further needed to gain more insights on the effect of DBS on swallowing function in movement disorders.

## Data Availability Statement

All datasets analyzed for this study are included in the article and the Table 1.

## Author Contributions

KT and TX initiated the study and made the study PRISMA compatible systematic review. HY, SQ, LB, and TX performed search and initial writeup. All authors edited and worked on the submitted manuscript.

## Conflict of Interest

The authors declare that the research was conducted in the absence of any commercial or financial relationships that could be construed as a potential conflict of interest.
